# Matrix metalloproteinase inhibition attenuates right ventricular dysfunction and improves responses to dobutamine during acute pulmonary thromboembolism

**DOI:** 10.1111/jcmm.12163

**Published:** 2013-11-06

**Authors:** Evandro M Neto-Neves, Ozelia Sousa-Santos, Karina C Ferraz, Elen Rizzi, Carla S Ceron, Minna M D Romano, Luis G Gali, Benedito C Maciel, Richard Schulz, Raquel F Gerlach, Jose E Tanus-Santos

**Affiliations:** a Department of Pharmacology, Faculty of Medicine of Ribeirao Preto, University of Sao PauloRibeirao Preto, Brazil; b Department of Pharmacology, State University of CampinasCampinas, Brazil; c Department of Internal Medicine, Medical School of Ribeirao Preto, University of Sao PauloRibeirao Preto, Brazil; d Departments of Pharmacology & Pediatrics, Cardiovascular Research Centre, University of AlbertaEdmonton, AB, Canada; e Department of Morphology, Estomatology and Physiology, Dental School of Ribeirao Preto, University of Sao PauloRibeirao Preto, Brazil

**Keywords:** pulmonary thromboembolism, doxycycline, matrix metalloproteinases, pulmonary hypertension, right ventricular dysfunction, cardiomyocyte injury

## Abstract

Activated matrix metalloproteinases (MMPs) cause cardiomyocyte injury during acute pulmonary thromboembolism (APT). However, the functional consequences of this alteration are not known. We examined whether doxycycline (a MMP inhibitor) improves right ventricle function and the cardiac responses to dobutamine during APT. APT was induced with autologous blood clots (350 mg/kg) in anaesthetized male lambs pre-treated with doxycycline (Doxy, 10 mg/kg/day, intravenously) or saline. Non-embolized control lambs received doxycycline pre-treatment or saline. The responses to intravenous dobutamine (Dob, 1, 5, 10 μg/kg/min.) or saline infusions at 30 and 120 min. after APT induction were evaluated by echocardiography. APT increased mean pulmonary artery pressure and pulmonary vascular resistance index by ∼185%. Doxycycline partially prevented APT-induced pulmonary hypertension (*P* < 0.05). RV diameter increased in the APT group (from 10.7 ± 0.8 to 18.3 ± 1.6 mm, *P* < 0.05), but not in the Doxy+APT group (from 13.3 ± 0.9 to 14.4 ± 1.0 mm, *P* > 0.05). RV dysfunction on stress echocardiography was observed in embolized lambs (APT+Dob group) but not in embolized animals pre-treated with doxycycline (Doxy+APT+Dob). APT increased MMP-9 activity, oxidative stress and gelatinolytic activity in the RV. Although doxycycline had no effects on RV MMP-9 activity, it prevented the increases in RV oxidative stress and gelatinolytic activity (*P* < 0.05). APT increased serum cardiac troponin I concentrations (*P* < 0.05), doxycycline partially prevented this alteration (*P* < 0.05). We found evidence to support that doxycycline prevents RV dysfunction and improves the cardiac responses to dobutamine during APT.

## Introduction

Acute pulmonary thromboembolism (APT) is a critical disorder causing pulmonary hypertension because of obstruction of pulmonary vessels by emboli and associated pulmonary vasoconstriction 1. Sudden elevation in right ventricular pressure overload may culminate in acute right ventricular dysfunction (RVD), circulatory shock and death 2–3. Indeed, haemodynamic stable patients with APT and RVD have poor prognosis and increased risk of death 4. Therefore, therapies targeting RVD secondary to APT may improve haemodynamics and survival.

Multiple pathological pathways have been proposed to influence the development of heart failure, including the activation of proteases in heart muscle 5. Particularly matrix metalloproteinases (MMPs), which are well known for their capacity to cleave extracellular matrix proteins, participate in myocardial remodelling during cardiac diseases 6–7. Recently, the intracellular actions of MMPs have been shown to also contribute to the development of acute heart failure 7,8. For example, MMP-2 may degrade sarcomeric and cytoskeletal proteins such as troponin I 10, myosin light chain-1 11, α-actinin 12 and titin 13. Supporting this idea, we have shown that increased MMPs were associated with cardiomyocyte injury following APT 14. However, no previous study has evaluated the possible role of MMPs in RVD during APT. While our previous study showed evidence for cardiomyocyte injury following APT 14, it is not known whether MMP inhibition results in less RVD during APT, and testing this possibility is critical to support further testing in future clinical trials. In addition, prevention of MMP-induced myocardial damage may reduce or preclude the need for therapeutic interventions including inotropic support with drugs such as dobutamine which aim at increasing RV contractility in the APT setting 15. It is also unknown whether MMP inhibition and protection against cardiomyocyte injury during APT improves right ventricular responses to inotropic support.

In this study, we suggest that pre-treatment with doxycycline (a broad-spectrum MMP inhibitor) would prevent RVD and improve cardiac responsiveness to dobutamine during experimental APT.

## Materials and methods

### Animal model and *in vivo* measurements

The study was approved by the Institutional Animal Care and Use Committee of the Faculty of Medicine of Ribeirao Preto, University of Sao Paulo, and the animals were handled according to the guiding principles published in the National Institutes of Health Guide for the Care and Use of Laboratory Animals.

Fifty-eight mixed-bred male lambs (21.4 ± 3.3 kg) were anaesthetized with ketamine (10–15 mg/kg, i.m.) and xylazine (0.1 mg/kg, i.m.), and relaxed with pancuronium (0.1 mg/kg, i.v.). Following tracheal intubation, they were mechanically ventilated with room air using a volume-cycled ventilator (C.F.Palmer, London, UK). The tidal volume was set at 12–15 ml/kg and the respiratory rate was adjusted to maintain physiological arterial carbon dioxide tensions 16. Anaesthesia was maintained with an intramuscular injection of ketamine (5 mg/kg) and midazolam (0.5–1 mg/kg) every 30 min. Fluid-filled catheters were placed into the left femoral artery and right femoral vein for mean arterial pressure monitoring *via* a pressure transducer and fluid and drug administration, respectively. A 7.5 F balloon-tipped pulmonary artery thermodilution catheter was placed into the pulmonary artery *via* the left femoral vein. The catheter was connected to pressure transducers to allow the monitoring of mean pulmonary artery pressure, central venous pressure and pulmonary artery occlusion pressure. Thermodilution cardiac output measurements were determined in triplicate by injecting 3 ml of saline and the results recorded (DX2010 Dixtal Monitor Dixtal, Dixtal do Brasil, Manaus, Brazil). The heart rate was measured using a surface electrocardiogram (lead I). A polyethylene catheter was inserted into the right ventricle *via* the right jugular vein to monitor right ventricular pressures, which were recorded using a data acquisition system (MP150CE; Biopac Systems Inc., Santa Barbara, CA, USA) connected to a computer (Acknowledge 3.2, for Windows). The first derivative of right ventricular pressure (dP/dt) was calculated and value of the maximum rate of isovolumic pressure development (dP/dt_max_) was used as index of contractility.

A venous blood sample (3 ml/kg) was collected and allowed to clot for at least 60 min., then cut into 2–3 mm cubes. In this study, APT was induced by infusing the autologous clots (350 mg/kg) for 15 min. *via* a large-bore cannula placed in the right atrium. This model of APT is very similar to that previously reported 14,17. After, at least 20 min. of stabilization, baseline haemodynamics were measured. The animals were randomly assigned to one of eight experimental groups as follows: a group of Sham operated, non-embolized animals were divided into four groups (*n* = 5–7 each): animals pre-treated with saline, which received saline (**1-Sham**) or increasing doses (1, 5, 10 μg/kg/min.; 5 min. for each dose at 30 and 120 min. after APT) of dobutamine (**2-Sham+Dob**); and animals pre-treated with doxycycline (10 mg/kg i.v.), which received saline (**3-Doxy**) or increasing doses of dobutamine (**4-Doxy+Dob**). Similarly, embolized animals were divided into four groups (*n* = 8–10 each): animals pre-treated with saline, which received saline (**5-APT**) or increasing doses (1, 5, 10 μg/kg/min.; 5 min. for each dose at 30 and 120 min. after APT) of dobutamine (**6-APT+Dob**); and animals pre-treated with doxycycline (10 mg/kg i.v.), which received saline (**7-Doxy+APT**) or increasing doses of dobutamine (**8-Doxy+APT+Dob**). This dose of doxycycline (Rhobifarma, Sao Paulo, Brazil) was chosen as the basis of previous studies showing the beneficial haemodynamic effects and MMPs inhibition 14–19. Dobutamine (Hipolabor, Sao Paulo, Brazil) was used to examine the cardiac responses to an inotropic drug and was infused as described below.

Haemodynamic evaluation was performed 60 min. before APT induction (baseline time-point) and then 0, 30, 60, 90 and 120 min. after APT induction. The cardiac index, systemic vascular resistance index and pulmonary vascular resistance index were calculated by standard formulae. Changes in cardiac index and dP/dt_max_ were calculated before and during the highest dose (10 μg/kg/min.) of dobutamine at 30 and 120 min. after APT. Arterial blood samples were collected at baseline and 120 time-point and serum samples were stored at −70°C. RV samples were collected and washed in cold saline, snap frozen and stored at −70°C.

### Evaluation of the RV by echocardiography

Echocardiographic examinations were performed in animals at baseline, 30 and 120 min. after APT (HD11XE Philips), with a S3 sectorial transducer. Images in the parasternal view (longitudinal and short axis at basal level and mid ventricular level) were acquired and digitally stored. Parasternal short axis view at the papillary level was used to measure the maximum diastolic linear dimension of the right ventricle (DDRV) and the systolic displacement of the lateral wall of the RV (SDRV) with M Mode technique. The global RV systolic function was subjectively evaluated taking into account the degree of wall dysfunction, assigning 1 point to normal function, 2 to slight dysfunction, 3 to moderate dysfunction and 4 to accentuated dysfunction. The stress echocardiography protocol consisted of increasing doses of intravenous dobutamine (1, 5 and 10 μg/kg/min., 5 min. for each dose), and echocardiography evaluation of myocardial systolic motion was performed during the highest dose at 30 and 120 min. after APT or saline infusion.

### Measurement of MMP-2 and MMP-9 in RV by SDS-PAGE gelatin zymography and gelatinolytic activity assay

To assess MMP-2 and MMP-9 in the RV, gelatin zymography was performed as previously described 14–20. Briefly, RV samples were subjected to electrophoresis on SDS-PAGE co-polymerized with gelatin (1%) as the substrate. Gelatinolytic activities were normalized with regard to an internal standard (foetal bovine serum) to allow intergel analysis and comparison. MMP-2 and MMP-9 were identified as bands at 72 and 92 kD, respectively.

Net MMPs activities in the RV were measured using a gelatinolytic activity kit (E12055, Molecular Probes, Eugene, OR, USA), as previously described 14,20.

### Measurement of MMP-2 protein levels by Western blot

Right ventricular samples were homogenized in ice-cold RIPA buffer. Fifty micrograms of protein extracts were subject to SDS-PAGE using a 12% polyacrylamide gel. The proteins were then transferred onto nitrocellulose membranes and blocked with TBST buffer (NaCl 100 mmol/l; Tris–HCl 100 mmol/l; Tween 0.1%) containing 5% bovine serum albumin. The membranes were incubated overnight at 4°C with anti-MMP-2 (1:1000, Chemicon, Billerica, MA, USA). Anti-β-actin (1:5000, Millipore, Billerica, MA, USA) was used as a loading control. The antibodies were washed in TBST and incubated with horseradish peroxidase (HRP)-secondary goat antimouse antibody (1:1000, Millipore) for 1 hrs. Immunolabelled proteins were visualized using chemiluminescence ECL (Millipore) and registered by ChemiDoc™ detection system (Bio-Rad, Hercules, CA, USA). The signal intensities were quantified using ImageJ Program (NIH —National Institute of Health).

### Measurement of MMP activities by *in situ* zymography

*In situ* MMPs activities were measured in frozen RV samples using DQ Gelatin (E12055, Molecular Probes) as a fluorogenic substrate. Briefly, RV samples were embedded in Tissuetek® and cut into 5 μm sections with a cryostat. Sample sections were incubated with 1.0 μg/ml DQ gelatin in Tris-CaCl_2_ buffer (50 mM Tris, 10 mM CaCl_2_, 1 μM ZnCl_2_) in dark humidified chambers for 1 hr. Negative control experiments were performed with the addition of 50 μM 1,10-phenanthroline to inhibit MMP activity. The sections were examined with fluorescent microscopy (Leica Imaging Systems Ltd., Cambridge, UK) and the image was captured at ×400. Proteolytic activity was detected as bright green fluorescence, which indicates substrate breakdown, and was evaluated by using ImageJ Program (NIH) 14–22.

### Assessment of oxidative stress in RV

Dihydroethidium (DHE), a fluorescent dye, was used to evaluate *in situ* production of reactive oxygen species (ROS) and reactive nitrogen species (RNS) production. Briefly, RV samples were embedded in Tissue-tek and then frozen and cut in serial 5 μm sections. Unfixed cryosections were incubated at room temperature, in the dark, with 30 μl of DHE (10 μmol/l in 0.01% dimethyl sulfoxide) for 30 min. Sections were examined by fluorescence microscopy (Leica imaging Systems Ltd.) and the image was captured at magnification of 400×. Red fluorescence produced by DHE oxidation to hydroxyethidium was evaluated by using Image J software (NIH) 14–22.

### Measurement of serum cardiac troponin I concentrations

Cardiac troponin I (cTnI) was measured in serum separated from arterial blood (centrifuged at 1100 × *g* at 37°C for 15 min.) with a commercially available kit (Vidas Troponin I Ultra-TNIU, Biomérieux, Durham, NC, USA) according to the manufacturer’s instructions. The detection limit for this assay is 0.01 μg/l of cTnI 20.

### Statistical analysis

The results are expressed as means ± SEM or are shown as dot and the median. Two-way (treatments X time) anova for repeated measures and Bonferroni post-test were used to compare haemodynamic and echocardiographic parameters. Biochemical parameters were compared between groups by one-way anova followed by the Dunnett multiple comparisons test. The global RV systolic function was compared between groups with Kruskall–Wallis test followed by Dunn’s *post hoc* test. A *P* < 0.05 was considered the minimum level of statistical significance.

## Results

### Pre-treatment with doxycycline ameliorated APT-induced pulmonary hypertension and minor increases in cardiac output and dP/dt_max_

**Figure 1:**
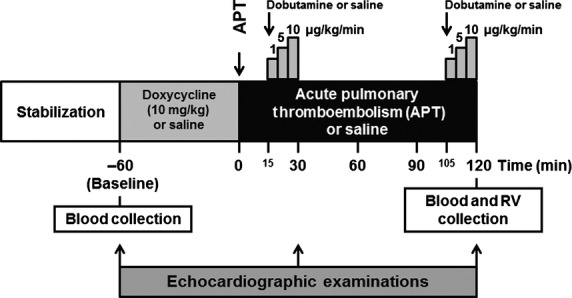
Schematic representation of the acute pulmonary thromboembolism (APT) protocol.

**Figure 2:**
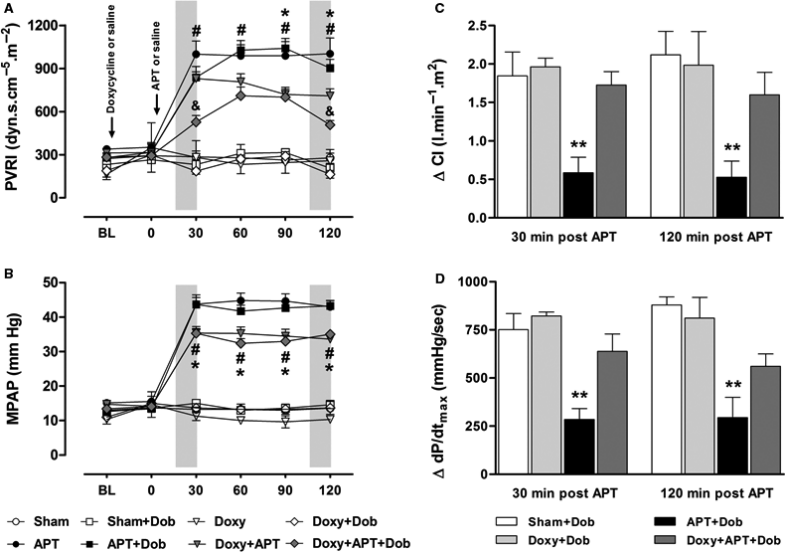
Pulmonary vascular resistance index (PVRI; A) and mean pulmonary arterial pressure (MPAP; B) at baseline (BL), and 0, 30, 60, 90 and 120 min. after acute pulmonary thromboembolism (APT) induction or saline infusion in Sham, Sham+Dob, Doxy, Doxy+Dob, APT, APT+Dob, Doxy+APT and Doxy+APT+Dob groups. Changes in cardiac index (CI; C) and maximum rate of isovolumic pressure development (dP/dt_max_; D) at 30 and 120 min. after APT induction or saline infusion in Sham+Dob, Doxy+Dob, APT+Dob and Doxy+APT+Dob (*n* = 5–10 per group). Values are the mean ± SEM. Grey bars indicate dobutamine infusion. *P < 0.05 APT *versus* Doxy+APT group. #*P* < 0.05 APT+Dob *versus* Doxy+APT+Dob group. **P < 0.05 APT+Dob *versus* others groups.

Schematic representation of the interventions performed is shown in Figure 1. Baseline haemodynamic parameters were similar in all experimental groups and showed no significant changes in Sham and Doxy groups throughout the study period (Fig. 2A and B, and Figs S1–S3). Conversely, APT significantly increased pulmonary vascular resistance index and mean pulmonary artery pressure (by ∼185%) in the APT group (Fig. 2A and B; both *P* < 0.05). The sustained pulmonary hypertension induced by APT in the APT+Dob group was associated with minor increases in cardiac index and RV dP/dt_max_ (but not in heart rate) produced by increasing doses of dobutamine at 30 and 120 min. after APT (Fig. 2C and D, Fig. S2A and S3; *P* < 0.05). While APT produced no significant changes in systemic vascular resistance index and mean arterial pressure (Fig. S1), it significantly increased heart rate and RV dP/dt_max_ in APT group by ∼75% and 110%, respectively (Figs S2B and S3; *P* < 0.05).

Pre-treatment with doxycycline (Doxy+APT group) was associated with lower increases in pulmonary vascular resistance index and mean pulmonary artery pressure (by ∼130% and 110%, respectively) compared with those found in the APT group, especially at the 120 time-point (Fig. 2A and B; both *P* < 0.05). Indeed, dobutamine infusion in the Doxy+APT+Dob group produced additional decreases in PVRI at 30 and 120 min. after APT induction (Fig. 2A; *P* < 0.05). Although minor increases in CI and dP/dt_max_ were observed after increasing doses of dobutamine in APT+Dob group, higher CI and dP/dt_max_ values were found in the Doxy+APT+Dob group at 30 and 120 min. after APT (Fig. 2C and D, Figs S2A and S3; *P* < 0.05).

### Doxycycline attenuates APT-induced RVD and dilatation

**Figure 3:**
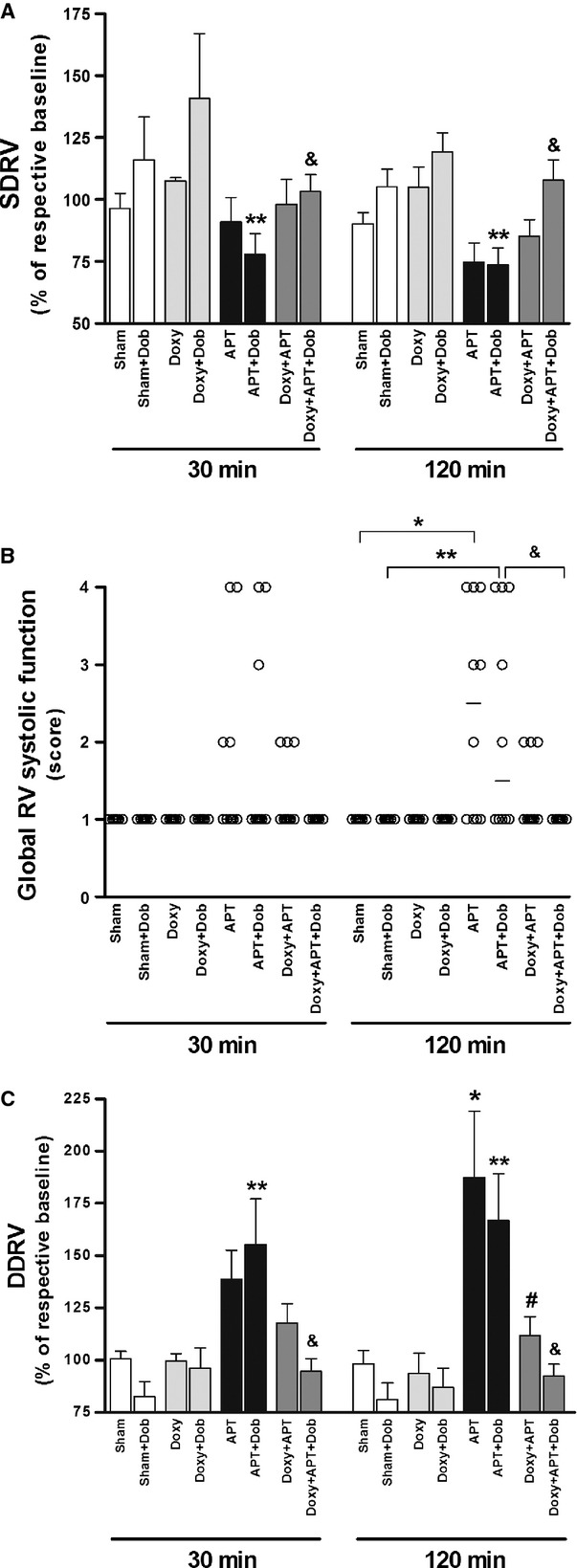
Percentage of respective baseline systolic displacement of the lateral wall of the RV (SDRV; A), global RV systolic function (B) and maximum diastolic linear dimension of the right ventricle (DDRV; C) measured by echocardiography at 30 and 120 min. after APT are shown for Sham, Sham+Dob, Doxy, Doxy+Dob, APT, APT+Dob, Doxy+APT and Doxy+APT+Dob groups (*n* = 5–10 per group). *P < 0.05 APT *versus* Sham group. **P < 0.05 APT+Dob *versus* Sham+Dob group. #*P* < 0.05 APT *versus* Doxy+APT group. &*P* < 0.05 APT+Dob *versus* Doxy+APT+Dob group.

Echocardiography was performed to evaluate RV function and diameter. A marked deterioration of RV function was observed in embolized animals stimulated with dobutamine (APT+Dob group) assessed by SDRV parameter (73 ± 7%) *versus* Sham+Dob group (105 ± 7%) especially at 120 min. after APT (Fig. 3A and Fig. S4; *P* < 0.05). Indeed, pre-treatment with doxycycline improved the right ventricular responses (evaluated by SDRV parameter) to increasing doses of dobutamine (107 ± 8%) in Doxy+APT+Dob group (Fig. 3A and Fig. S4; *P* < 0.05). While embolized animals (APT and APT+Dob groups) developed RVD (confirmed by global RV systolic function), pre-treatment with doxycycline (Doxy+APT+Dob group) protected against RVD following APT (Fig. 3B; *P* < 0.05). The maximum diastolic linear dimension of the right ventricle increased by >150% in the APT and in the APT+Dob groups (Fig. 3C and Fig. S5; *P* < 0.05). Interestingly, pre-treatment with doxycycline prevented RV dilatation induced by APT both in the Doxy+APT and in the Doxy+APT+Dob groups (Fig. 3C and Fig. S5; *P* < 0.05).

### Doxycycline causes MMPs inhibition, and prevents oxidative stress and cardiomyocyte injury

**Figure 4:**
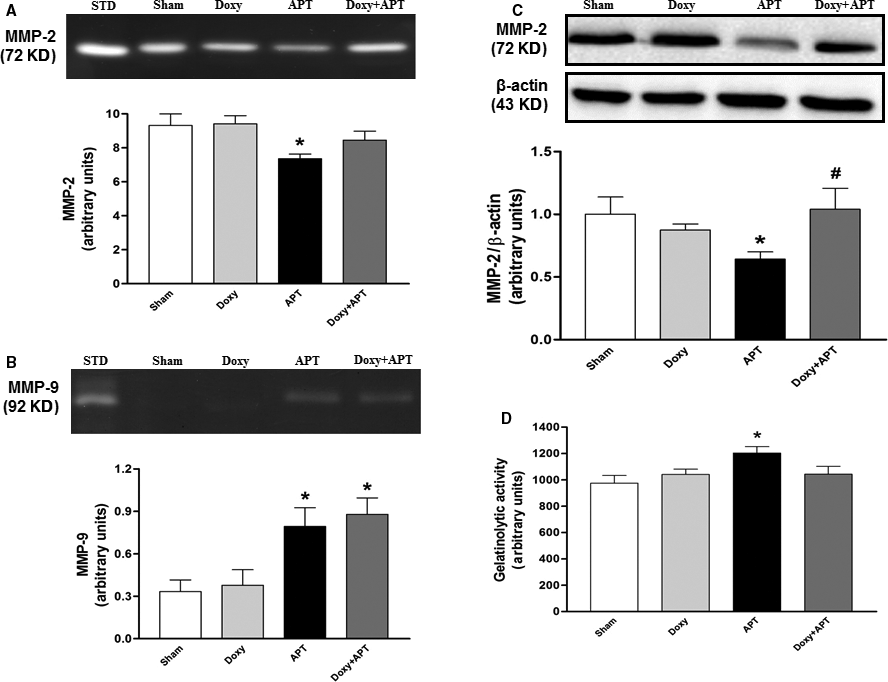
Representative sodium dodecyl sulphate-polyacrylamide gel electrophoresis gelatin zymogram of right ventricle samples showing the bands corresponding to MMP-2 (72 kD) and MMP-9 (92 kD), the densitometric data for MMP-2 and MMP-9 are showed in A and B respectively. (C) Representative Western blotting gel showing the expression of RV MMP-2 (72 kD), densitometric intensity corresponding to each band was normalized using β-actin (43 kD) expression. Net gelatinolytic activity in RV measured with a gelatinolytic activity kit (D) and the assays described above are shown for Sham, Doxy, APT and Doxy+APT groups (*n* =5–10 per group). STD: internal standard. Values are the mean ± SEM. *P < 0.05 *versus* Sham group. #*P* < 0.05 APT *versus* Doxy+APT group.

Acute pulmonary thromboembolism was associated with a loss in RV MMP-2 activity/protein (measured by zymography and western blot, respectively; Fig. 4A and C; *P* < 0.05), and with increase in RV MMP-9 activity (Fig. 4B; *P* < 0.05). Importantly, a loss in tissue MMP-2 level reflects its precedent activation 23, whereas increased MMP-9 likely reflects neutrophil migration. Although doxycycline produced no changes in RV MMP-9 activity (Fig. 4B), it significantly prevented the loss of RV MMP-2 protein measured by western blot (Fig. 4C; *P* < 0.05). In parallel with these findings, the zymography assay showed similar trends to those found with western blot (Fig. 4A). In addition, the measurement of net MMP gelatinolytic activity in RV samples showed that APT increased RV gelatinolytic activity by ∼25% in the APT group, but not in the Doxy+APT group (Fig. 4D; *P* < 0.05).

**Figure 5:**
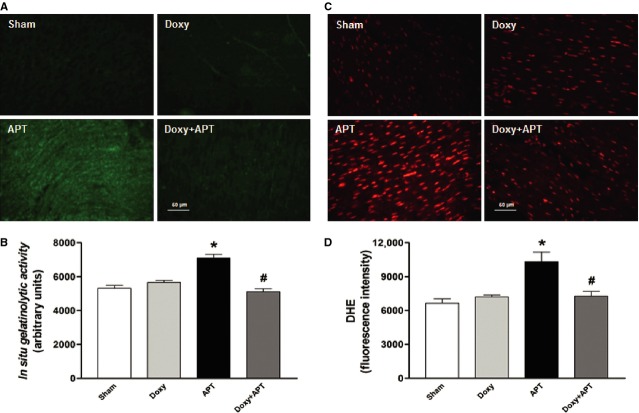
Representative photomicrographs of *in situ* gelatinolytic activity (A) and DHE oxidation (C) in the right ventricle cryosections from Sham, Doxy, APT and Doxy+APT groups. The quantification of green (gelatinolytic activity) and red fluorescence intensity (DHE oxidation) for each group (*n* = 5–10 per group) are show in panels B and D, respectively. Values are the mean ± SEM. *P < 0.05 APT *versus* Sham group. #*P* < 0.05 APT *versus* Doxy+APT group.

Figure 5 shows green fluorescence (which indicates proteolytic activity; Fig. 5A) and red fluorescence which indicates ROS/RNS production (Fig. 5C) in representative fluorescence photomicrographs of right ventricular cryosections. The analysis of *in situ* zymography and DHE oxidation confirmed that APT increased MMP activity by >30% and ROS/RNS production by >50% in the RV of embolized animals, respectively. Doxycycline prevented these increases (*P* < 0.05; Fig. 5B and D, respectively). In addition, 1,10-phenantroline attenuated increased gelatinolytic activity in RV samples evaluated by *in situ* zymography (data not shown).

**Figure 6:**
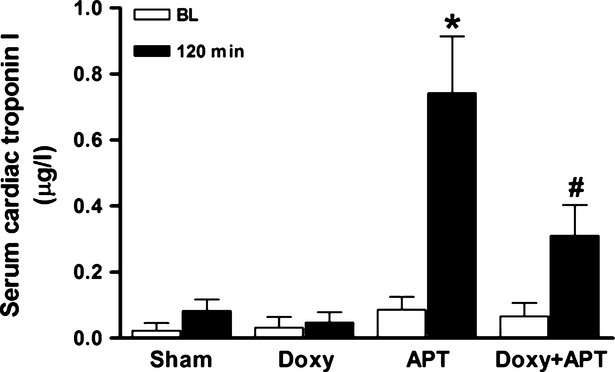
Serum cardiac troponin I concentrations at baseline (white bars) and 120 min. (black bars) after APT or saline infusion in Sham, Doxy, APT and Doxy+APT groups (*n* = 5–10 per group). Values are the means ± SEM. *P < 0.05 *versus* respective BL. #*P* < 0.05 APT *versus* Doxy+APT group at 120 min.

To assess cardiomyocyte injury after APT, we measured cTnI release into the blood (Fig. 6). Serum cTnI concentrations increased at least ninefold at 120 min. after APT (Fig. 6; *P* < 0.05), and doxycycline partially prevented this increase (Fig. 6; *P* < 0.05).

## Discussion

This is the first study to show that doxycycline (a broad-spectrum MMP inhibitor) prevents RVD and improves cardiac responsiveness to dobutamine after APT. Furthermore, doxycycline protects against APT-induced dilatation. While our previous study showed that doxycycline protects against cardiomyocyte injury during APT, this is the first study to clearly show that MMP inhibition prevents against RVD caused by APT. The beneficial haemodynamic effects were associated with lower overall gelatinolytic activity, and ROS/RNS production, as well as with reduced cTnI release. Together, these findings are consistent with a protective effect by doxycycline against cardiomyocyte injury associated with MMP activation during APT, and clearly show for the first time that MMP inhibition prevented the impairment in RV function during this critical condition.

In the clinical setting, RVD predicts poor outcome and it is commonly observed in patients with APT. While echocardiography is considered the reference standard for the assessment of RVD, cardiac troponins have emerged as possible cardiac biomarkers and have been used in combination with echocardiography for risk stratification in patients with pulmonary embolism 24. In line with clinical findings, we observed RVD and dilatation associated with increased serum cTnI after experimental APT. In fact, cTnI release may be explained by APT-induced cardiomyocyte damage 25 and serum cTnI concentrations increase in proportion to the degree of APT severity 26. Importantly, we found that doxycycline prevented RVD and dilatation, probably as result of protection against cardiomyocyte injury (revealed by serum cTnI concentrations) 27. These beneficial effects could be of great clinical relevance to patients at high risk who present RVD after APT.

Inotropic support may be used to increase RV contractility in patients with right heart failure 28. However, inotropic stimulation may not adequately improve right ventricular function under particular conditions 29. It is clear that MMPs activation contributes to APT-induced myocardial damage, thus worsening cardiac responsiveness to positive inotropes in the APT setting. Supporting this idea, we found minor increases in cardiac contractility parameters (on echocardiography and haemodynamics) in embolized animals stimulated with dobutamine, whereas MMP inhibition with doxycycline improved the responses to dobutamine during APT. Therefore, MMPs inhibitors such as doxycycline may protect the heart, especially the RV, against myocardial damage and improve cardiac performance following APT. Our findings suggest that doxycycline could be used in combination with inotropic agents to increase cardiac contractility during APT.

Elevated pulmonary vascular resistance leading to ischaemia of the RV has been considered the major determinant of APT-induced RVD 2–30. However, right ventricular inflammation has also recently been shown to contribute to RVD and raised levels of circulating cardiac biomarkers in experimental 31,32 and human fatal cases of pulmonary embolism 34–35. Although the exact mechanism by which inflammatory responses contribute to RVD remains unknown, it is possible that inflammatory cells (neutrophils and macrophages) release pre-stored proteinases including MMP-9^36^, thus contributing to RVD following APT. In agreement with this suggestion, we and others have shown evidence implicating MMP-9 in APT-induced pulmonary hypertension and cardiomyocyte injury 14–38. In this study, we found increased MMP-9 levels and gelatinolytic activity in RV after APT. Doxycycline prevented the increases in RV gelatinolytic activity, and this effect was associated with protective effects against RVD and dilatation, as well as cTnI release following APT. Taken together, our results reinforce the idea that MMP-9 up-regulation and MMP activation promote myocardial injury and contribute to APT-induced RVD and dilatation.

In this study, we showed for the first time a loss in RV MMP-2 protein/activity that was accompanied by increased gelatinolytic activity in RV during APT. Furthermore, doxycycline prevented both the loss of RV MMP-2 levels and increased RV gelatinolytic activity. In agreement with these findings, hearts subjected to ischaemia and reperfusion were shown to release MMP-2, and the pharmacological inhibition of MMPs protected against ischaemia and reperfusion-induced myocardial injury 23. Although pro-MMP-2 may be activated by proteolytic mechanisms, non-proteolytic MMP activation produced by oxidant stress has recently been reported 39. Supporting this suggestion, the antioxidant drug tempol decreased RV MMP up-regulation/activation following APT 20. Therefore, it is possible that increased oxidative stress observed in RV after APT promotes both RV MMP activation and cardiac cell damage, thus contributing to loss of MMP-2 from RV, as we observed in this study. Indeed, it is possible that antioxidant effects exerted by doxycycline 40 may contribute to the protective effects reported here.

Emerging evidence now shows that MMPs may target intracellular structures resulting in cardiac dysfunction 7,8. In particular, MMP-2 degrades cardiac proteins including troponin I 10, myosin light chain-1^11^, α-actinin 12, and titin 13, and impairs cardiac responsiveness to dobutamine 21. Here, we observed increased cTnI release into serum following APT, which suggest cTnI degradation in the hearts of embolized animals. Interestingly, doxycycline partially prevented the increases in serum cTnI, probably as a result of both MMP inhibition and antioxidant effects together. In agreement with our findings, MMP-2 has been shown to cleave troponin I, both *in vitro* and *in vivo*, and this effect was prevented by MMP inhibitors 10. Therefore, cardiac protein degradation induced by MMP activation could play a major role in cardiomyocyte injury and subsequent RVD after APT. Supporting this idea, our results show that doxycycline-mediated MMP inhibition partially prevented cTnI release in association with improved right ventricular function during APT, which may be consequence of both intracellular and extracellular cardiac effects 21.

This study has some limitations. We used doxycycline as an MMP inhibitor in this study. This drug is non-specific and may have non-MMP effects. It would be interesting to test selective MMP inhibitors whenever they are available. On the other hand, doxycycline is an approved drug that has been used for decades in clinical Medicine 41. Another limitation is that we examined the effects of a pre-treatment with doxycycline. While our study design is powerful to identify a role for MMPs in the RVD, it would be interesting to test doxycycline as a rescue treatment. It is important to mention that part of the beneficial haemodynamic effects observed here may be a consequence of doxycycline-reduced RV afterload. Therefore, to confirm our findings, would be interesting to evaluate the role of MMP inhibition in a pressure non-dependent model of RV failure. Finally, we have observed increased haemolysis in our experimental model of APT probably as a consequence of pulmonary regurgitation and lysed red blood cells from the delivered clots 42. Importantly, haemolysis has been associated with pulmonary vasoconstriction, which may contribute to increased oxidative stress. Therefore, it is possible that MMP activation may be, at least in part, explained by APT-induced haemolysis.

In conclusion, doxycycline prevents RVD and RV dilatation, and improves the cardiac responses to dobutamine during APT. Our findings suggest that MMP inhibition may protect against a proteolytic deleterious profile that impairs cardiac function during this critical condition.

## Supporting Information

Figure S1. Systemic vascular resistance index (SVRI; A) mean arterial pressure (MAP; B) at baseline (BL), and 0, 30, 60, 90 and 120 min. after APT induction or saline infusion in Sham, Sham+Dob, Doxy, Doxy+Dob, APT, APT+Dob, Doxy+APT and Doxy+APT+Dob groups (n = 5–10 per group). **Figure S2.** Cardiac index (CI; A) and heart rate (HR; B) at baseline (BL), and 0, 30, 60, 90 and 120 min. after APT induction or saline infusion in Sham, Sham+Dob, Doxy, Doxy+Dob, APT, APT+Dob, Doxy+APT and Doxy+APT+Dob groups (n = 5–10 per group).
