# Supplementary material for: Matrix metalloproteinase inhibition attenuates right ventricular dysfunction and improves responses to dobutamine during acute pulmonary thromboembolism
Source: J Cell Mol Med. 2013 Nov 6;17(12):1588–97. doi: 10.1111/jcmm.12163 (PMC3914650; doi:10.1111/jcmm.12163)
Supplement: Supplementary file 1 — Figure S1. Systemic vascular resistance index (SVRI; A) mean arterial pressure (MAP; B) at baseline (BL), and 0, 30, 60, 90 and 120 min. after APT induction or saline infusion in Sham, Sham+Dob, Doxy, Doxy+Dob, APT, APT+Dob, Doxy+APT and Doxy+APT+Dob groups (n = 5–10 per group). Figure S2. Cardiac index (CI; A) and heart rate (HR; B) at baseline (BL), and 0, 30, 60, 90 and 120 min. after APT induction or saline infusion in Sham, Sham+Dob, Doxy, Doxy+Dob, APT, APT+Dob, Doxy+APT and Doxy+APT+Dob groups (n = 5–10 per group). [file jcmm0017-1588-sd1.doc]

**SUPPLEMENTAL MATERIAL**


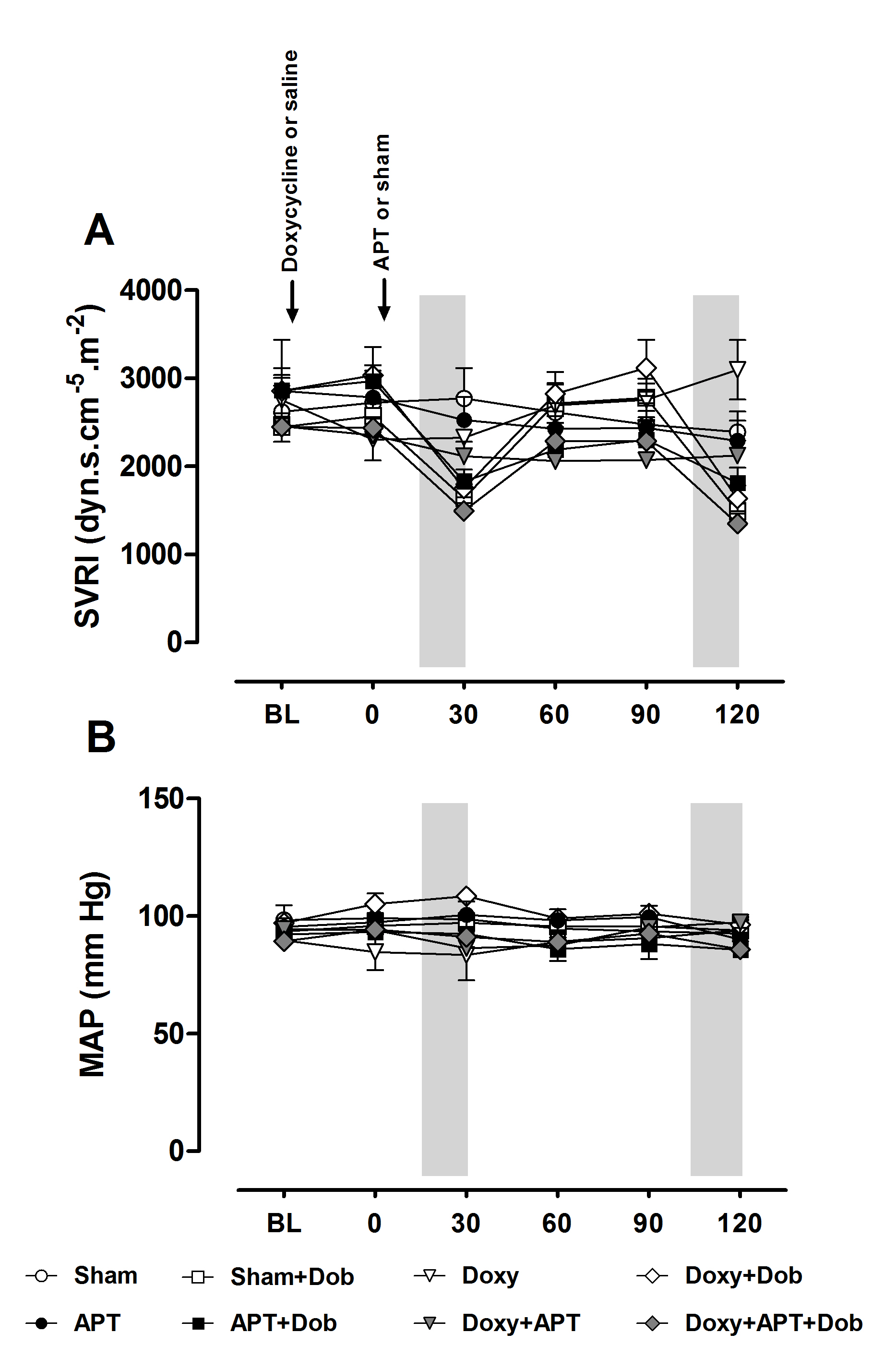
**Supplemental figures and figure legends**

**Figure S1:** Systemic vascular resistance index (SVRI; Panel A) mean arterial pressure (MAP; Panel B) at baseline (BL), and 0, 30, 60, 90, and 120 min after APT induction or saline infusion in Sham, Sham+Dob, Doxy, Doxy+Dob, APT, APT+Dob, Doxy+APT, and Doxy+APT+Dob groups (n=5-10 per group).

Values are the mean + S.E.M.

Gray bars indicate dobutamine infusion


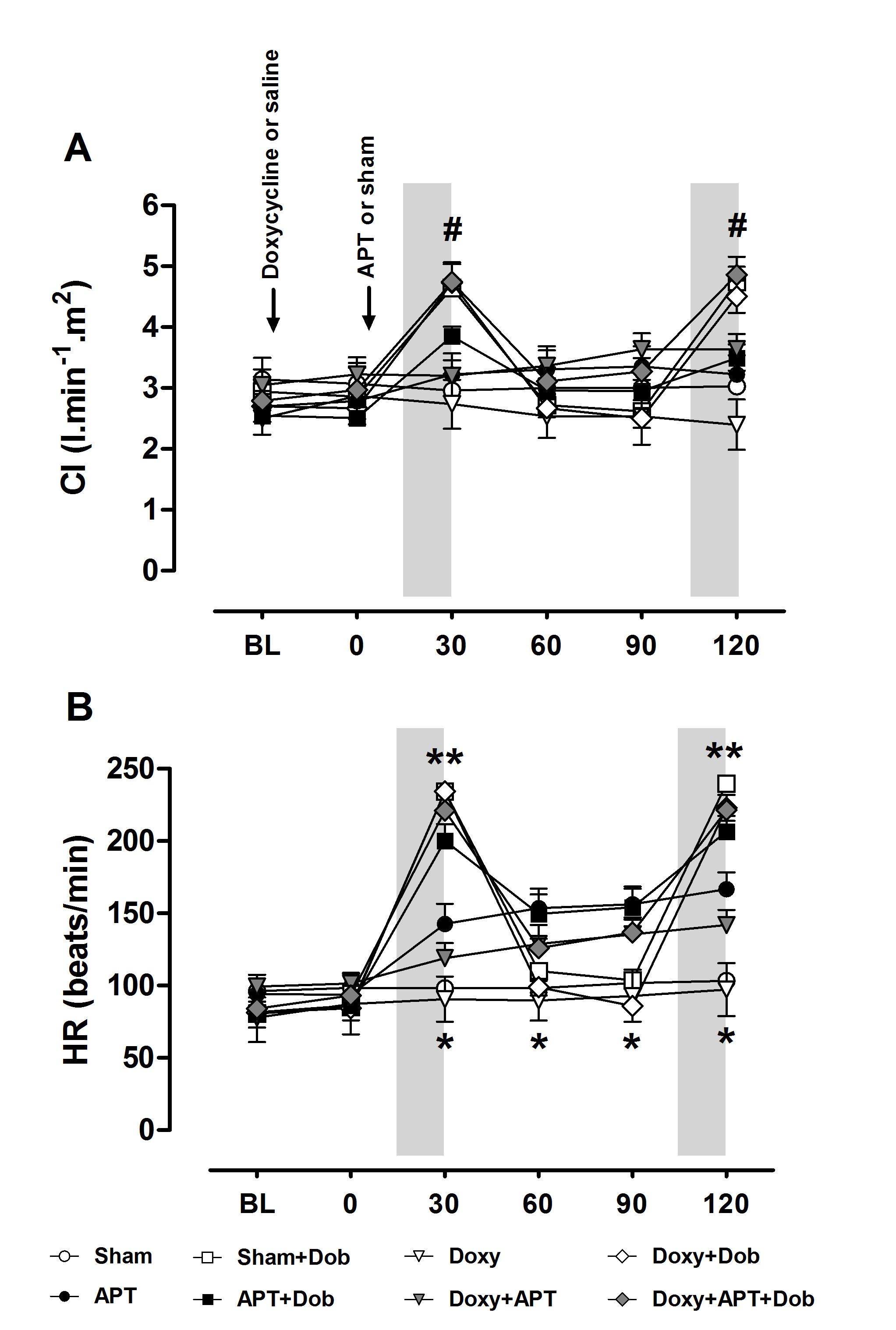


**Figure S2:** Cardiac index (CI; Panel A) and heart rate (HR; Panel B) at baseline (BL), and 0, 30, 60, 90, and 120 min after APT induction or saline infusion in Sham, Sham+Dob, Doxy, Doxy+Dob, APT, APT+Dob, Doxy+APT, and Doxy+APT+Dob groups (n=5-10 per group).

Values are the mean + S.E.M.

Gray bars indicate dobutamine infusions.

^*^ p< 0.05 APT *versus* Sham group.

^**^ p< 0.05 Sham *versus* Sham+Dob, Doxy *versus* Doxy+Dob, APT *versus* APT+Dob, and Doxy+APT *versus* Doxy+APT+Dob.

^#^ p< 0.05 APT+Dob *versus* Doxy+APT+Dob.


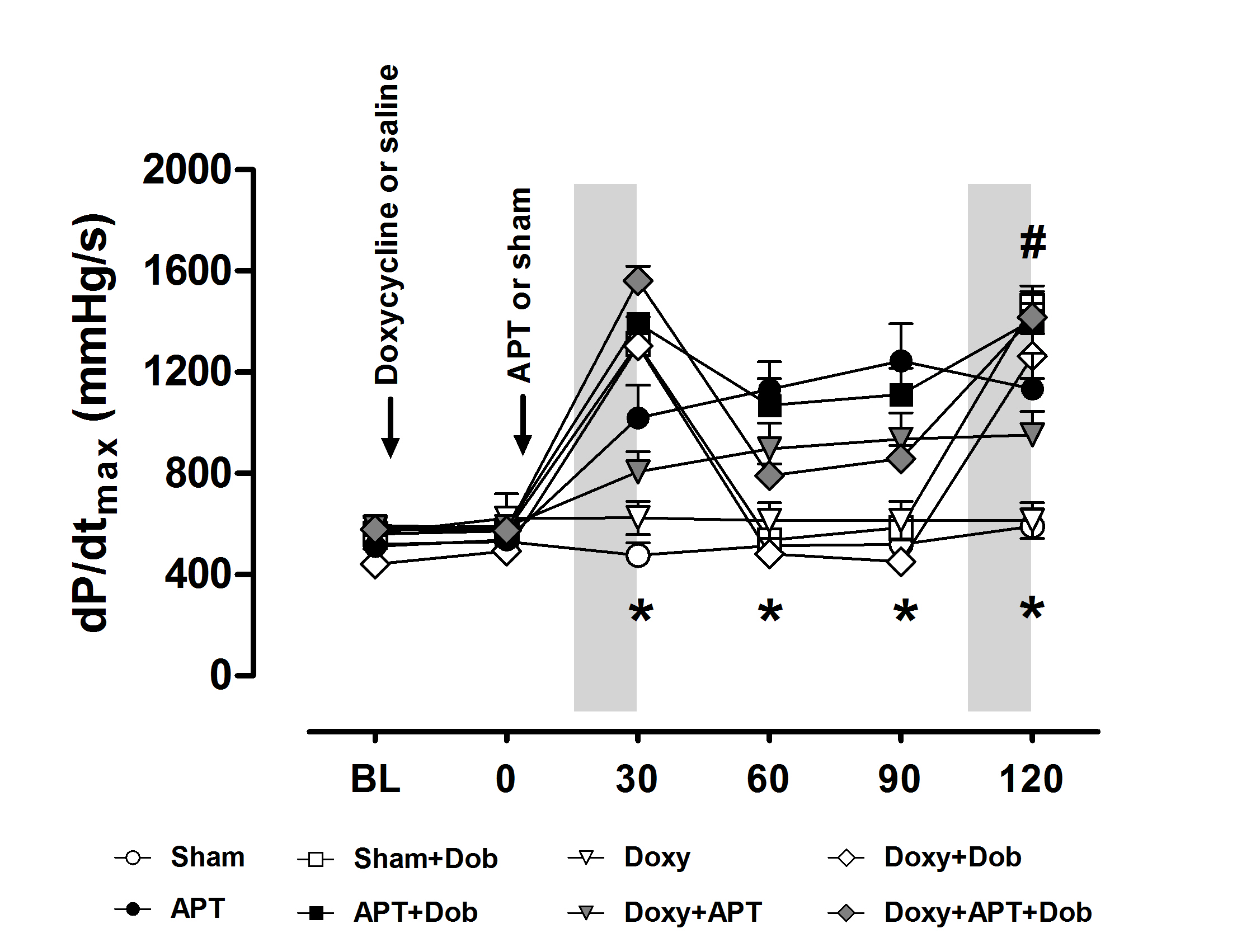


**Figure S3.** Maximum rate of isovolumic pressure development (dP/dt_max_ ) at baseline (BL), and 0, 30, 60, 90, and 120 min after APT induction or saline infusion in Sham, Sham+Dob, Doxy, Doxy+Dob, APT, APT+Dob, Doxy+APT, and Doxy+APT+Dob groups (n=5-10 per group).

Values are the mean + S.E.M.

Gray bars indicate dobutamine infusions.

^*^ p< 0.05 APT *versus* Sham group.

^#^ p< 0.05 Sham *versus* Sham+Dob, Doxy *versus* Doxy+Dob, and Doxy+APT *versus* Doxy+APT+Dob.


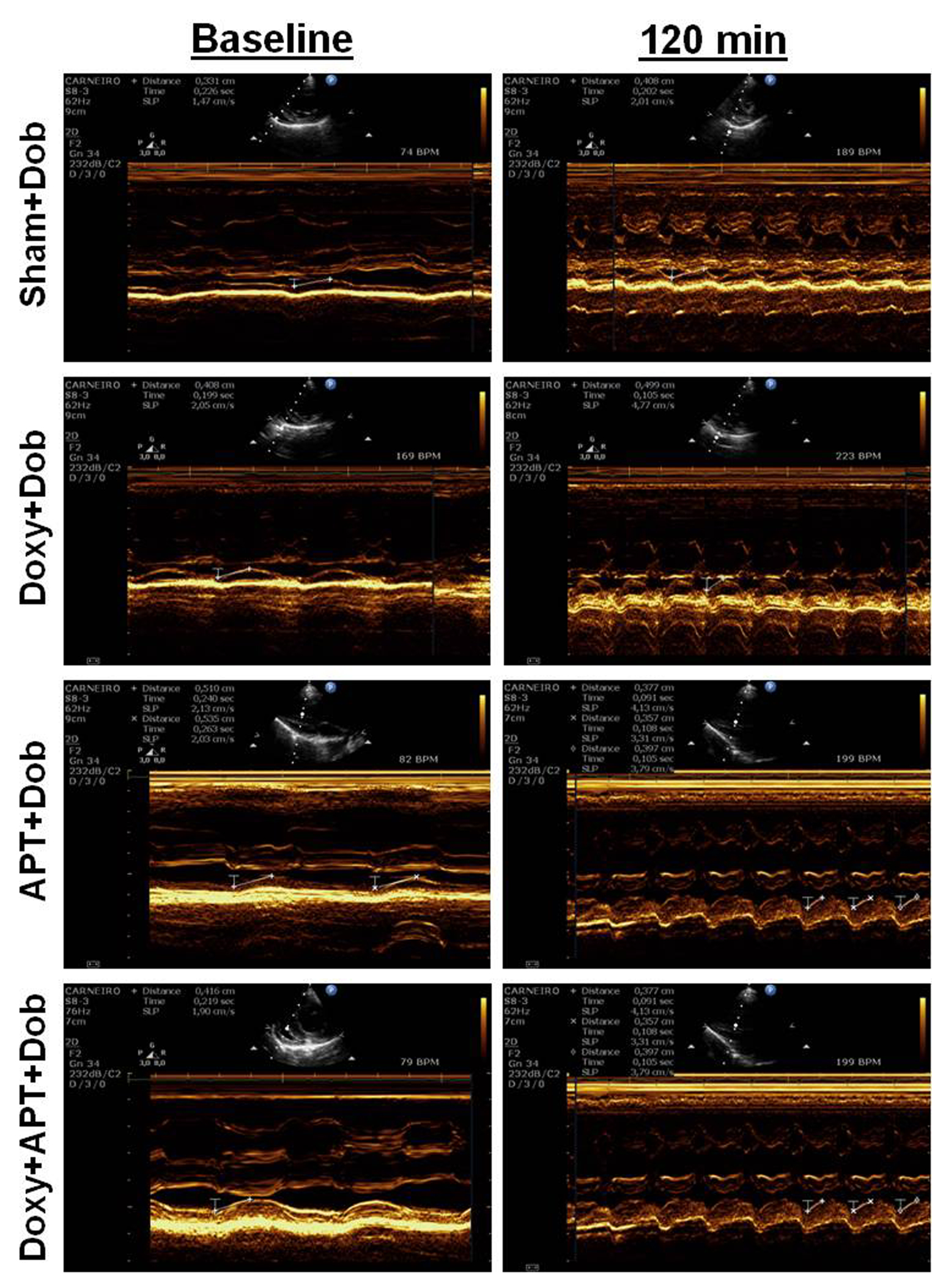


**Figure S4.** Representative photographs obtained from echocardiography (M Mode technique) used to measure the systolic displacement of the lateral wall of the RV (SDRV) at BL and 120 min after APT induction or saline infusion in Sham+Dob, Doxy+Dob, APT+Dob and Doxy+APT+Dob groups.


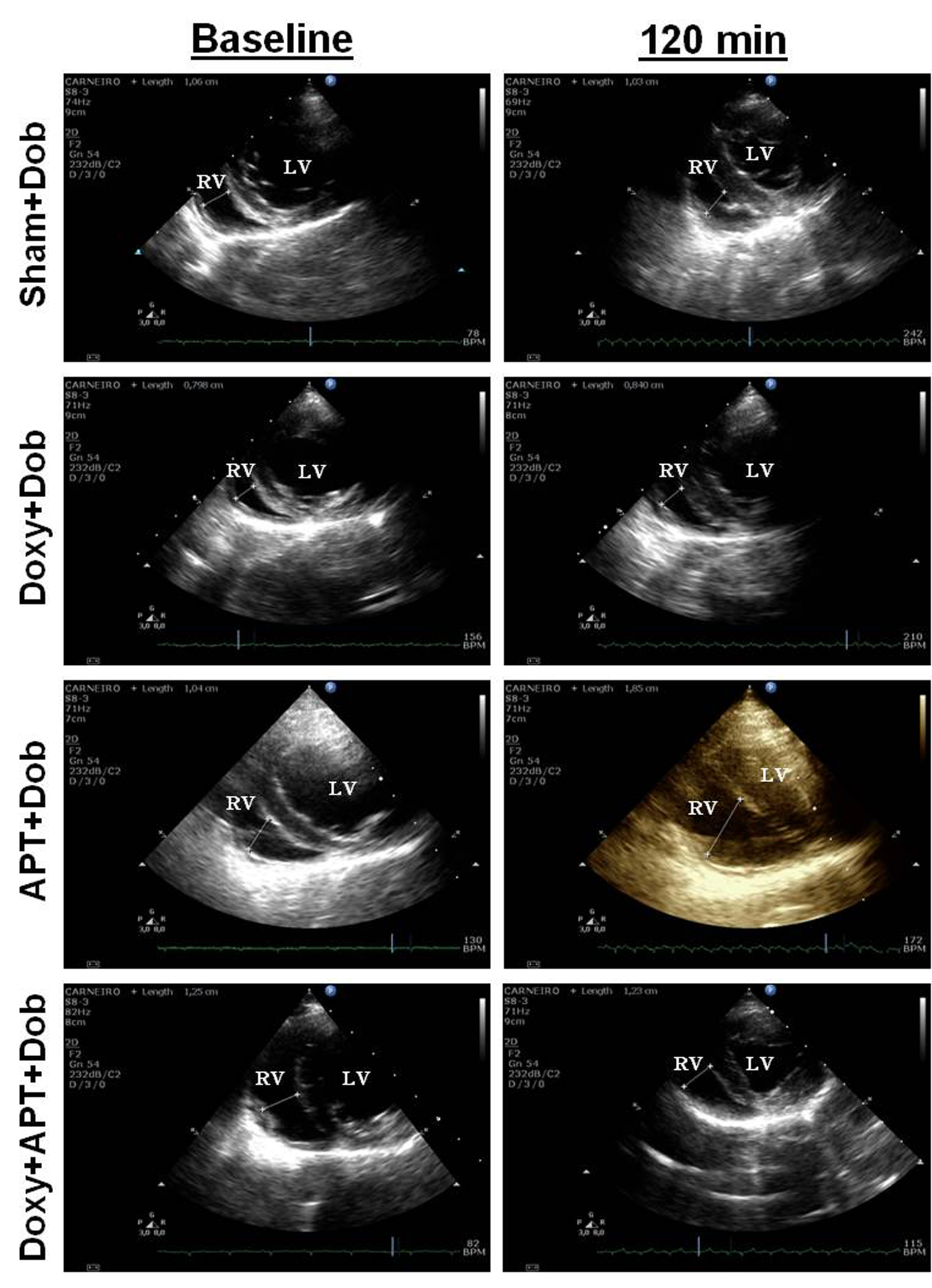


**Figure S5.** Representative photographs obtained from echocardiography used to measure the maximum diastolic linear dimension of the right ventricle (DDRV) at BL and 120 min after APT induction or saline infusion in Sham+Dob, Doxy+Dob, APT+Dob and Doxy+APT+Dob groups.
